# Non-invasive volumetric ultrasound localization microscopy detects vascular changes in mice with Alzheimer's disease

**DOI:** 10.7150/thno.99097

**Published:** 2025-01-01

**Authors:** Rebecca M. Jones, Ryan M. DeRuiter, Mohanish Deshmukh, Paul A. Dayton, Gianmarco F. Pinton

**Affiliations:** 1 Joint Department of Biomedical Engineering, University of North Carolina at Chapel Hill and North Carolina State University, Chapel Hill, NC, 27599, USA.; 2 Neuroscience Center, University of North Carolina at Chapel Hill, Chapel Hill, NC, 27599, USA.

**Keywords:** volumetric ultrasound, non-invasive imaging, super-resolution imaging, Alzheimer's Disease, microvascular assessment

## Abstract

Alzheimer's Disease (AD) is the most common form of dementia and one of the leading causes of death. AD is known to be correlated to tortuosity in the microvasculature as well as decreases in blood flow throughout the brain. However, the mechanisms behind these changes and their causal relation to AD are poorly understood.

**Methods:** Here, we use volumetric ultrasound localization microscopy (ULM) to non-invasively and quantitatively compare the microvascular morphology and flow dynamics of five wildtype (WT) and five APP^NL-G-F^ Knock-in mice, a mouse model of AD, across a 1cmx1cmx1cm brain volume and in four specific brain regions: the hippocampal formation, thalamus, hypothalamus, and cerebral cortex.

**Results:** Comparisons between groups showed a significant increase in tortuosity, as measured by the Sum of Angles Metric (SOAM), throughout the brain (p < 0.01) and the hypothalamus (p = 0.01), in mice with AD. While differences in mean velocity (p < 0.01) and blood flow (p=0.04) were detected across the whole brain, their effect size was small and no differences were detected in the four selected regions. There was a significant decrease in the linear log relationship between vessel diameter and blood flow, with AD mice experiencing a lower slope than WT mice across the whole brain volume (p = 0.02) and in the hippocampal formation (p = 0.05), a region affected by Amyloid Beta plaques in this mouse model. The AD mice had higher blood flows in smaller vessels and smaller blood flows in larger vessels than the WT mice.

**Conclusions:** This preliminary demonstrates that the imaging technique can be used for non-invasive, longitudinal, volumetric assessment of AD, which may allow for investigation into the poorly understood microvascular degeneration associated with AD through time as well as the development of early diagnostic techniques.

## Introduction

Alzheimer's Disease (AD) is a debilitating neurodegenerative brain disorder that impairs cognitive function. It currently affects 6.5 million Americans and is the 7th leading cause of death 1, 2. AD causes changes in the brain including brain atrophy and dysfunction. In humans, one of the first regions in the brain affected by Alzheimer's disease is the mnemonic system, composed of the hippocampus, entorhinal cortex, and thalamus 3, 4. The buildup of Amyloid Beta (A*β*) plaques is one of the predominant signs of AD and could contribute to brain degeneration. It has been shown that A*β* plaque formation begins in the thalamus, mammillary bodies, and parietal cortex as well as the temporal and occipital lobes 4, 5. This is then followed by the entorhinal cortex, hippocampus, and insular cortex 5. Vascular abnormalities including increased tortuosity, string vessel formation, bulging, and stenosis, have been observed in histopathology of AD 6. In addition, AD has been shown to cause increases in blood viscosity 7, 8 and vessel stiffness 9. Several methods that cause this vascular degradation have been studied 10-12; however, the relationship between these mechanisms and AD is poorly understood. Further investigation into the effects of AD on microvascular pathology are needed to better understand this phenomenon and to establish cause relationships between AD and vascular degeneration.

Ultrasound Localization Microscopy (ULM) is a rapidly emerging option for *in vivo* microvascular imaging being developed by several groups world-wide 13. This technique has been used to image the microvasculature in the brain of rodents 14, 15 and humans 16. A recent 2D super-resolution ultrasound imaging study performed in AD mice with a craniotomy demonstrated a significant reduction in blood velocity, an increase in vascular tortuosity in the whole brain and a reduction in blood volume in the cerebral cortex 17, 18. That study demonstrates the capability of this technique to study brain vasculature, particularly in AD mice. However, 2D super-resolution ultrasound imaging, even in conjunction with mechanical scanning, which requires long scan times, is limited by its inability to super-resolve transverse vessels, reducing its ability to characterize the vascular morphology, underestimating velocity, and total flow volume 19. Volumetric imaging is thus an essential component of microvascular imaging. Volumetric super-resolution has been recently demonstrated *in vivo* in transcranial rat and mouse brain vessels with spatial resolutions between 20-35 *µ*m in diameter and velocities between 2 mm/s to 100 mm/s 19-21. Here, we have non-invasively performed volumetric ULM through an intact skull and scalp on five wildtype (WT) mice and five mice with AD to compare differences between their vascular structure. Quantitative analysis of vessel morphology and hemodynamics demonstrate the strength of this imaging method and its potential for larger longitudinal studies that are powered to determine causal relationships between AD and vascular degradation as well as early diagnostic techniques.

## Methods

### Mouse model

For this study, APP^NL-G-F^ Knock-in mice were used, an AD mouse model that forms cortical and sub-cortical A*β* plaques beginning at two months due to the over-expression of A*β* precursor protein (APP), experiencing amyloidosis in the hippocampus as well as subcortical regions 22, 23. This model mimics AD symptoms in humans, experiencing memory loss beginning at 6 months, reduction in spatial memory by 8 months, and reduction in object recognition by 10 months 24. In this mouse model, A*β* plaques begin to form at 2 months of age, becoming widespread by 7 months 22, 23. For this study, one male and four female mice with AD between 9 and 10 months of age were compared to three female and two male WT mice, between the ages of 7 and 11 months.

### Experimental setup

To perform volumetric ULM on mice, anesthesia was induced in each of the mice using 5% isoflurane gas carried by medical air, maintained at 2.5% throughout the experiment. Each of the mice were depilated, with both the skull and scalp remaining intact, and placed in a stereotaxic frame (Stoelting Co., Wood Dale, IL). Each animal was imaged with 1 cm between the top of the head and the transducer surface, coupled using ultrasound gel. A 1024-channel Verasonics Vantage (Verasonics, WA) system was used with a 7.81 MHz matrix array transducer (Vermon S.A., Tours, France), transmitting with a Mechanical Index (MI) of 0.5, as measured at the transducer surface, before attenuation through the skull and tissue. The system was calibrated to adjust for misalignment in the matrix array elements due to manufacturing 25. In-house fabricated microbubbles (MBs) 26, 27 were infused through a catheterized tail vein, using a syringe pump (Harvard Apparatus, Holliston, MA), at 3 *×* 10^7^ MB*/*mL*/*g, using a rate of 6 *µ*L*/*min. The infusion was started 2 minutes before data acquisition to allow for the microbubble concentration in the blood to reach a steady state. Data was acquired for 200 seconds with microbubbles infusing throughout. Five angled plane waves steered to *±* 3*^◦^* laterally or *±* 3*^◦^* elevationally were acquired at a pulse repetition frequency of 2500 frames per second, resulting in an effective volumetric frame rate of 500 volumes per second. Data was acquired at 2 points per wavelength (ppw) and filtered back up to 4 ppw, with the minimum depth required to capture the whole head saved. Each acquisition consisted of 492 GB of raw data, resulting in 4.9 TB across all ten animals. All experiments were performed in accordance with and with approval from the University of North Carolina at Chapel Hill Institutional Animal Care and Use Committee (IACUC). The experimental setup is shown in Fig. S1.

### Ultrasound localization microscopy

ULM volumes were created as previously described in McCall et al 21. Images were beamformed using delay and sum beamforming with a grid step size of *λ*/2. Then, the beamformed images were filtered using singular value decomposition (SVD) filtering to remove portions of the signal that decorrelate slowly, spatio-temporally, like the skull, leaving the portions that decorrelate quickly, like microbubbles 28. Images were scaled by depth to ensure consistent detection throughout the image. The microbubbles were then localized using weighted centroids and tracked using the Hungarian Algorithm (*simpletracker*) 29, creating a static image of the brain vasculature. Volumetric images were visualized using 3D Slicer 30 and 2D Maximum Intensity Projections (MIPs) were visualized using MATLAB (MathWorks, Natick, MA, USA). The resolution of the image was estimated using Fourier shell correlation (FSC) 31, a 3D version of Fourier Ring correlation 32, which determines the resolution across the image. All mice were processed using the same parameters.

### Mouse brain atlas registration

The brain was segmented into its different regions based on manual registration with a mouse brain atlas 33. This atlas allows for the alignment of the brain based on the specific shape and location of the brain for each mouse, accounting for differences between datasets. For this analysis, four specific regions, the hippocampal formation, thalamus, hypothalamus, and cerebral cortex, four of the large brain regions associated with AD progression within our field of view, were highlighted in the analysis.

### Microvascular analysis

Each of the ULM images were skeletonized to create a binary image of the vascular centerlines. Skeletonization was performed by first using Otsu's method to normalize the intensity of the vessels throughout the image. Then, the vessel centerlines were extracted using a GPU-based centerline extraction technique 34. This skeletonization was then used in the calculation of vessel average velocity, diameter, blood flow, density, and tortuosity metrics. Diameter was calculated by overlaying the skeletonized version of the ULM image on top of a binarized version of the ULM image and determining the least number of pixels from the center line to the edge of the vessel, then doubling it. Vessel density was estimated by calculating the percent of the brain filled with vasculature based on the binarized image. Tortuosity of the vessel structure was calculated using the established sum of angles metric (SOAM) 35-37. Velocity was calculated using the tracked microbubbles through time and space. The mean velocity at each point through time was calculated to create an overall velocity map. Then, the maximum velocity was extracted at the central points of each vessel. Assuming parabolic laminar flow, the average velocity over the vessel cross sectional area was taken as 1/2 of the maximum velocity. Average blood flow was then calculated by multiplying the average velocity and cross-sectional area of the vessel, calculated using the diameter, at each tracked point along the vessel. As vessel diameter increases, so does the blood flow. This relationship is described by the generalized extension of Murray's Law, where there should be a linear power law relationship between the two 20, 38. The power law slope was calculated for the relationship between diameter and blood flow for each of the mice.

### Statistical analysis

Comparisons were performed between the two groups using a Kruskal-Wallis one-way ANOVA for the diameter, velocity, blood flow, tortuosity, vessel density, and the power law slope between diameter and blood flow. For diameter, velocity, blood flow, and tortuosity, these comparisons were performed by comparing the average values for each vessel across the brain regions for all of the animals, separated by group. For the vessel density and power law slope between diameter and blood flow, these values contain only one value per animal. Data are displayed using violin plots, where blue represents the WT data and pink represents the AD data. Outliers are defined as data points that are 1.5x the inter-quartile range above the third quartile and below the first quartile and are represented by circles on the plots. Significant differences are represented with a star. For all comparisons, an alpha value of *α*=0.05 was selected for significance and the effect size was determined using Cohen's d. These comparisons were performed across the brain volume and across the four selected brain regions, segmented from the atlas. Statistical analysis was performed using MATLAB. All *p*-values and effect sizes are reported in Table S1.

## Results

### Ultrasound localization microscopy

**Figure 1:**
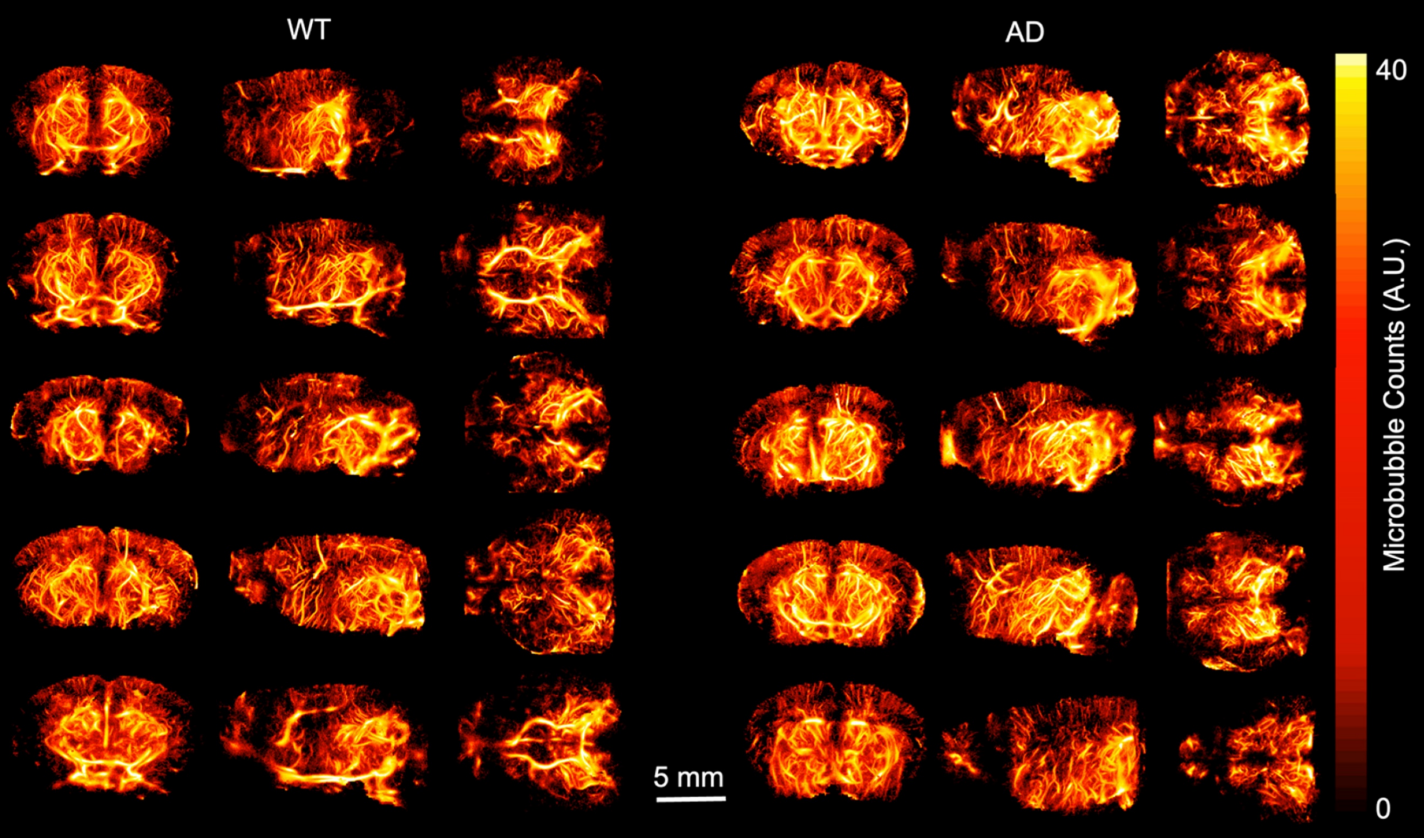
ULM volumetric renderings are shown for each of the ten mice including in the study, with views from the coronal, sagittal, and transverse dimensions shown. The ULM images were created by accumulating all of the microbubble tracks for each mouse, and for display, gaussian smoothing and power compression were used. Each image in plotted using the same colorbar axis, where microbubble tracks are in arbitrary units. All five WT mice are shown on the left and all five AD mice are shown on the right. Each volume is masked to include only the brain based on the registration to the Allen brain atlas. All of the images were created using 3dslicer.

**Figure 2:**
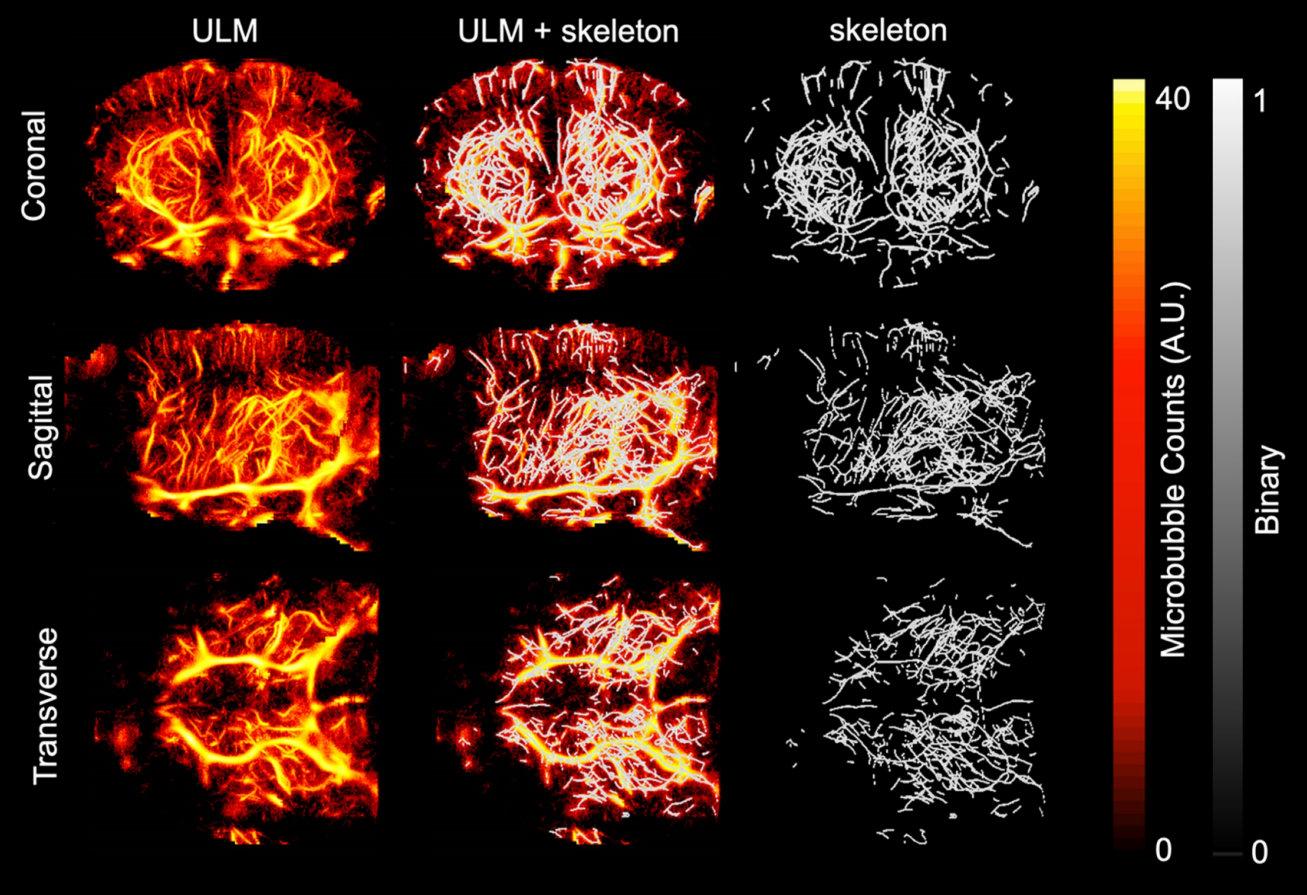
Maximum intensity projections (MIPs) of the smoothed and power compressed ULM image and its corresponding skeleton, calculated using Otsu's method for binarization followed by a gpu-based centerline extraction, are shown in each dimension. The first row corresponds to MIP collapsed in the coronal dimension, second to the MIP in the sagittal dimension, and third in the transverse. The first column shows the MIP of the ULM image. To allow for direct comparison with the resulting skeleton, the second column shows the skeleton MIP overlaid on the ULM MIP. The third column shows just the skeleton MIP. This example is using the second WT mouse in Fig. 1. The MIPs for all three dimensions are plotted using the same colorbar.

ULM images were created and analyzed for each of the ten mice included in the study. Volumetric renderings, created using 3dSlicer, are shown for the power compressed ULM image for each of the ten mice in Fig. 1. The WT mice are shown on the left and AD mice on the right. The coronal (1st column), sagittal (2nd column), and transverse (3rd column) views are shown for each mouse. Data is displayed in microbubble counts, with all of the renderings displayed with the same display settings. Each rendering is masked with the brain atlas to remove noise outside the brain. Across the ten mice, the region with the least number of visible vessels is the cortex, which is likely due to shadowing from the skull. All images had a resolution between 26.2 and 31.4 *µ*m, as measured by FSC. For each of the ULM images, the skeletonization was used to perform microvascular analysis. An example of a ULM image compared to the corresponding skeleton is shown in Fig. 2. The MIPs in the coronal (1st row), sagittal (2nd row), and transverse (3rd row) dimensions are shown. On the left, the power compressed ULM MIPs are shown for the second WT mouse in Fig. 1. Then, an overlaid image of the ULM MIP and the corresponding skeleton MIP are shown. The MIP of just the skeleton is shown on the right. While the skeleton does not detect all of the vessels in the cerebral cortex, the deeper regions are well represented.

### Blood flow

**Figure 3:**
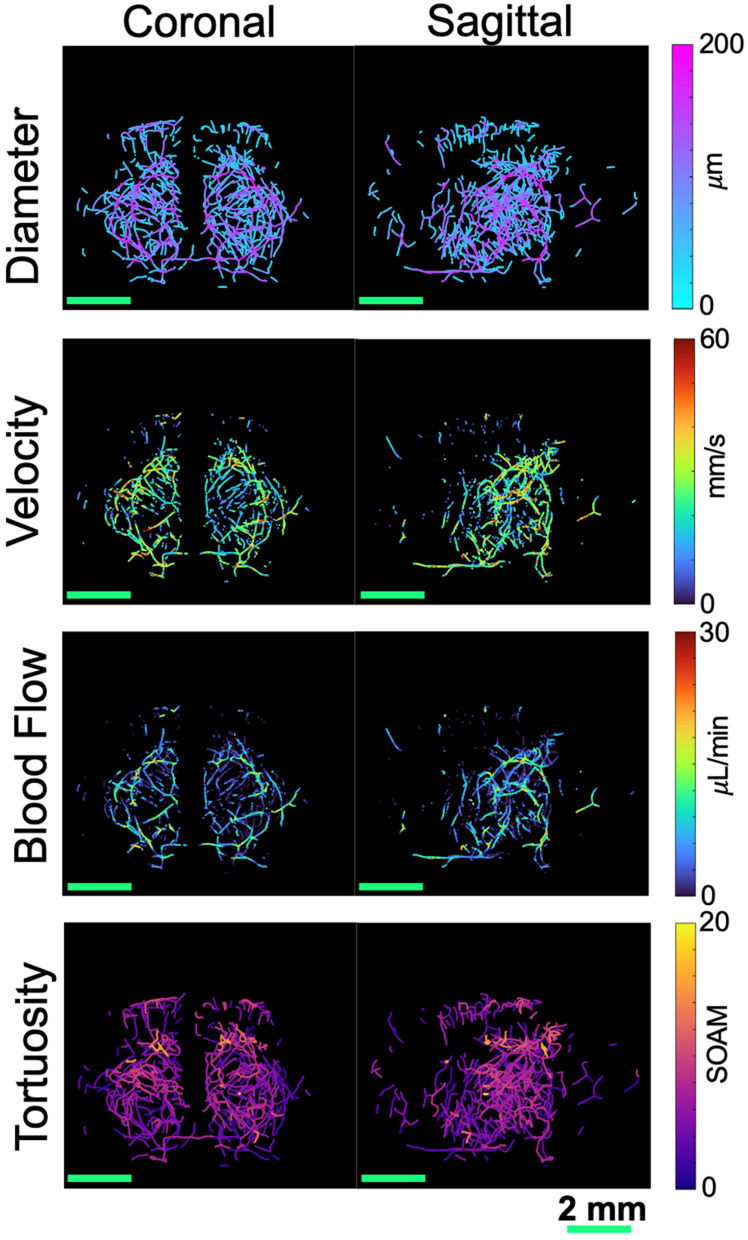
MIPs are shown for four different metrics, collapsed in both the coronal (left) and sagittal (right) dimensions for the second WT mouse from Fig. 1. The diameter, shown in the first row, was calculated based on the distance from the centerline to the closest edge in the binarized image. The velocity, shown in the second row, was calculated based on the average velocity at each centerline location from the tracked and localized data. These two parameters were then used to calculate the blood flow, shown in the third row. The tortuosity, shown in the fourth row, was calculated based on the sum of angles metric in each vessel. All scale bars are 2 mm.

**Figure 4:**
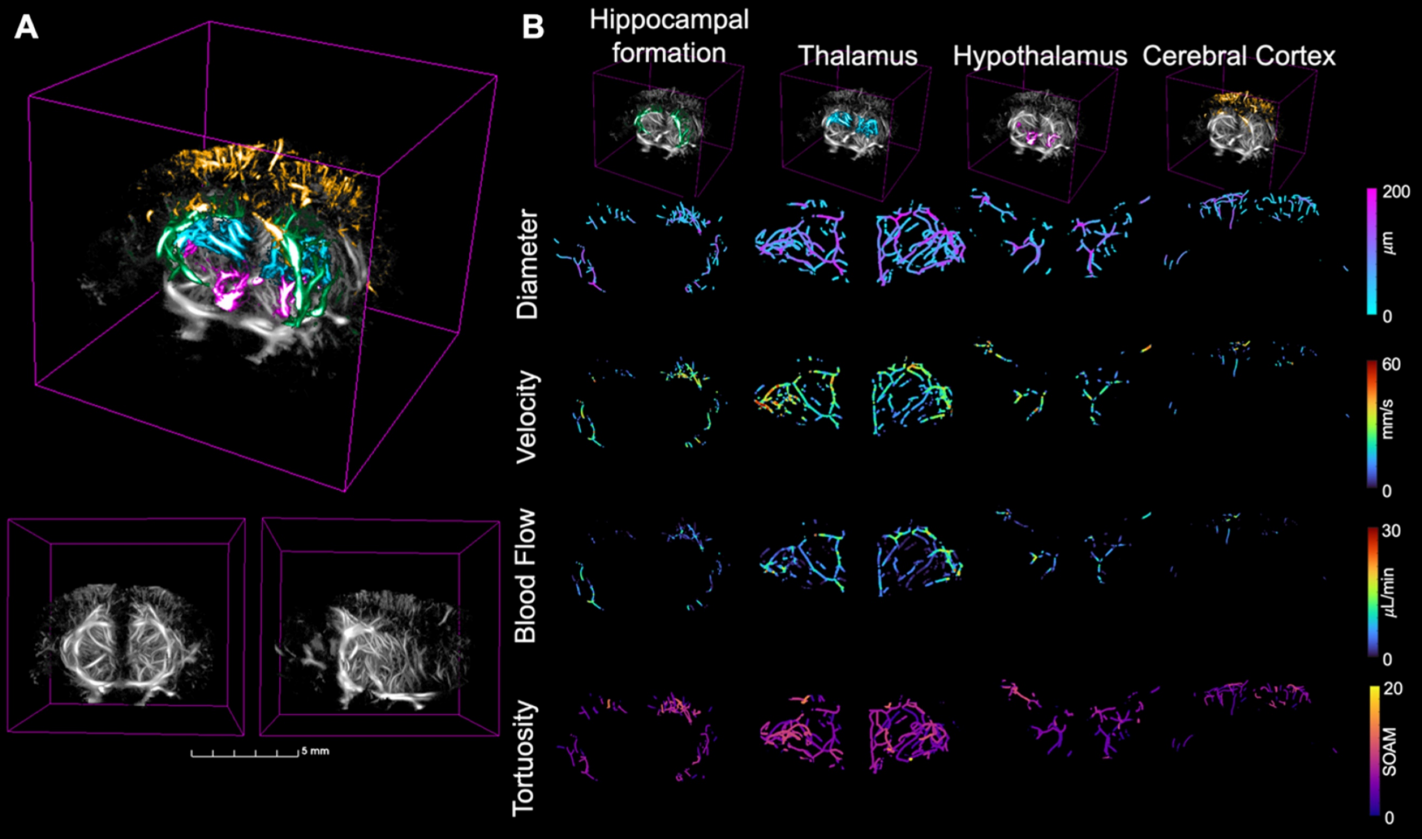
(A) The four brain regions of interest plotted over a grayscale ULM image where the green region is the hippocampal formation, blue the thalamus, pink the hypothalamus, and orange the cerebral cortex for the second WT mouse from Fig. 1. The coronal (left) and sagittal (right) views of the grayscale ULM image are shown below for comparison. (B) The zoomed in MIPs for each of the four compared metrics are calculated in each of the four regions, extracted from the skeleton based on the atlas registration. The regions are displayed across the top, with the hippocampal formation in the first column, thalamus in the second, hypothalamus in the third, and cerebral cortex in the fourth. The grayscale ULM image with just the corresponding region is shown in the top row. Then, the four metrics are shown over the skeleton masked for the four regions of interest based on the atlas registration. Diameter is first, followed by velocity, then blood flow, and finally tortuosity. The metrics are displayed on the same color axis for all four brain regions.

To analyze the resulting ULM images, the extracted skeletons were used to calculate vascular metrics including vessel density, diameter, velocity, and blood flow. These metrics were compared between the two groups across the brain volume and the four regions of interest, masked using the atlas registration. MIPs across the coronal and sagittal dimensions showing the diameter, velocity, and blood flow, projected onto the skeletonization of the ULM image are shown in Fig. 3, with the first row corresponding to diameter, second to velocity, and third to blood flow. Each mouse had vessel diameters between 19 and 170 *µ*m, velocities within the range of 0-50 mm/s, and blood flows between 0.005 and 20 *µ*L/min, with no significant differences between groups. These values are consistent with the literature for vasculature in the brains of mice 20. A 3D rendering of the four selected brain regions is shown in Fig. 4A, where a grayscale ULM image is overlaid by the four regions, with the hippocampal formation in green, thalamus in blue, hypothalamus in pink, and cerebral cortex in orange. The coronal and sagittal views of the grayscale ULM image are shown below the overlaid image for comparison. The MIPs for the evaluated metrics, projected onto the masked skeleton, for the four selected regions, are shown in Fig. 4B. For the diameter, larger vessels are located deeper in the brain, particularly in the thalamus. Smaller diameters are located within the cerebral cortex. Similar trends are seen for velocity and blood flow, where lower values are detected in the cerebral cortex and hypothalamus than the thalamus and hippocampus formation.

**Figure 5:**
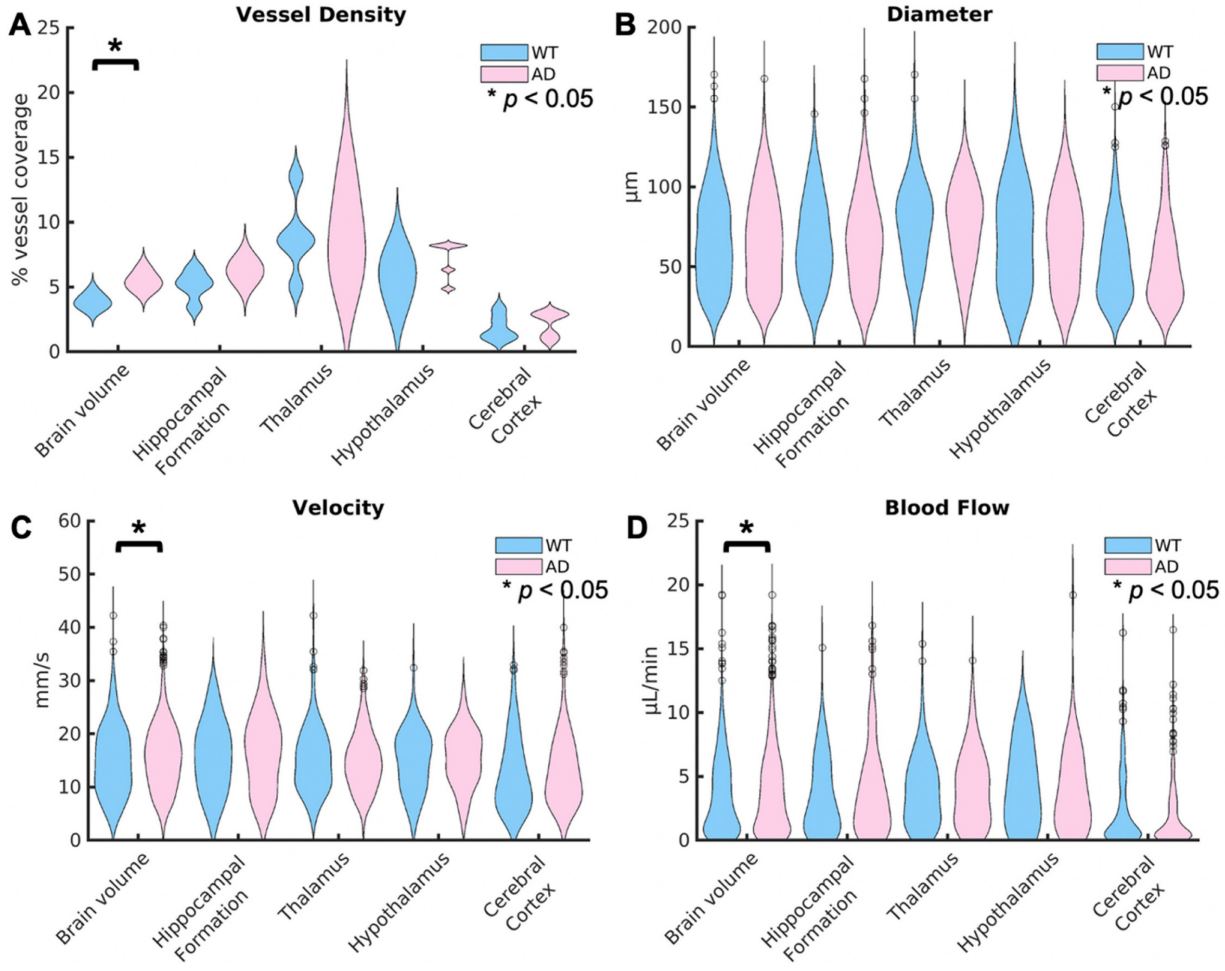
Violin plots are displayed for the vessel density for each mouse (A), the diameter per vessel (B), average velocity over the total acquisition time (C), and blood flow (D) for the WT and AD mouse groups. The distributions show the combined values for all of the mice in each group. The values across the brain volume are shown on the left, followed by the four selected regions of interest. For each region, the WT distribution is shown first in blue, followed by the AD distribution in pink. Statistical significance (*α* = 0.05) is represented by a *, with significance calculated using Kruskal-Wallis one-way ANOVAs. The vessel density, velocity, and blood flow were significantly higher in AD mice than WT mice across the brain volume but not any of the selected brain regions. There were no significant differences in vessel diameter between groups.

Violin plots showing the distributions for each of these metrics are shown in Fig. 5, with the distribution for WT mice shown first in blue, followed by AD mice shown in pink. In the top left, Fig. 5 A shows the comparisons between groups for the vessel density. Across the brain volume, AD mice (5.5% *±* 0.7%) have a significantly higher vessel density (p = 0.02), defined as the percent of vessel coverage from the binarized image, than WT mice (3.7% *±* 0.5%). This difference has an effect size, calculated using Cohen's d, of 2.5339, suggesting a very strong relationship. However, there are no significant differences in the other regions of interest. In Fig. 5 B, the violin plots for the diameter in *µ*m are shown for the two groups. There are no significant differences detected in diameter. In Fig. 5 C, the velocity across every vessel is shown. Similarly to the vessel density, AD mice (15.6 *µ*m *±* 6.2*µ*m) had a significantly higher (p = 0.002) velocity than WT mice (15.0 *µ*m *±* 6.5*µ*m) across the brain volume but no differences in the other selected regions. However, the comparison across the brain volume has a low effect size (d=0.1). This means that there is only a weak relationship in velocity. Fig. 5 D shown the blood flow between groups. Again, there is a significantly higher blood flow in mice with AD (3.8 *µ*L/min *±* 3.2*µ*L/min) than WT mice (3.5 *µ*L/min *±* 3.0*µ*L/min) across the brain volume (p = 0.04), but no differences in the regions of interest. However, this comparison again has a low effect size (0.09). The lack of differences in the selected brain regions compared to the whole brain volume could be due to the smaller number of vessels in those regions, limiting the power, or other regions could be driving this difference.

**Figure 6:**
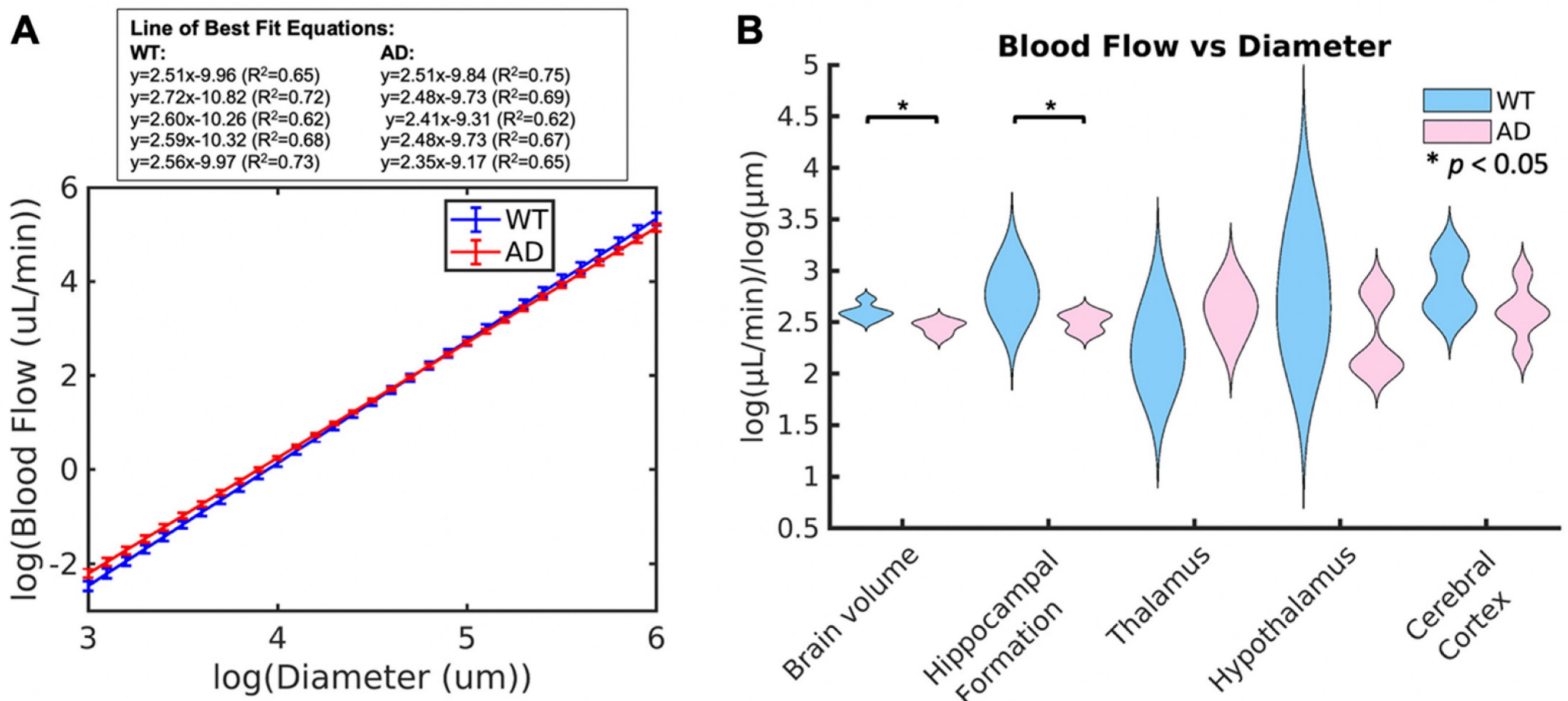
(A) Average line of best fit for the linear power law relationship between diameter and blood flow across the whole brain volume in WT (blue) and AD (red) mice. Error bars are shown, calculated based on the slope of each of the five mice. The equations for the lines of best fit for the ten mice are shown in the box above the plot. (B) Whisker plots showing the mean power law slope between diameter and blood flow across the whole brain volume and in each of the four selected regions for WT and AD mice are shown. Statistical significance (*α* = 0.05) is determined based on Kruskal-Wallis one-way ANOVAs and represented by *. The slope in AD mice was significantly lower than WT mice across the brain volume and in the hippocampal formation.

To determine if blood flow is consistent with respect to vessel diameter, the generalized extension of Murray's Law was applied. This law states that, based on vessel branching, there should be a linear log relationship between vessel diameter and blood flow. Based on this relationship, the slope should be between 2.4 and 3 for blood flow, with the ideal being around 2.5 38. WT mice had values ranging between 2.51 and 2.72 (2.60 *±* 0.08), which are within the expected values for a vascular network, while AD mice had values ranging between 2.35 and 2.52 (2.45 *±* 0.07), which are below the expected value range. The plots comparing the lines of best fit between groups are shown in Fig. 6 A. The average slope for each of the two groups is shown, with the error bars representing the differences within the group. The lines of best fit for each of the ten animals is shown in the box above the plot. From this plot, it can be seen that AD mice have higher velocities in smaller vessels but lower velocities in larger vessels than WT mice. In smaller and larger vessels, there is a separation between the two groups, but they overlap in the middle diameter range. The violin plots for this slope across the two groups is shown in Fig. 6 B. When comparing the groups, mice with AD have a significantly lower linear log relationship between blood flow and vessel diameter than WT mice across the brain volume (p = 0.02) and the hippocampal formation (p = 0.05, WT = 2.79 *±* 0.22, AD = 2.50 *±* 0.09). For both of these comparisons, the effect size is large, with an effect size of d = -2.0426 across the brain volume and d = -1.7234 in the hippocampal formation. Both of these effect sizes suggest a strong difference between the groups. There are no significant differences in the remaining brain regions.

### Tortuosity

**Figure 7:**
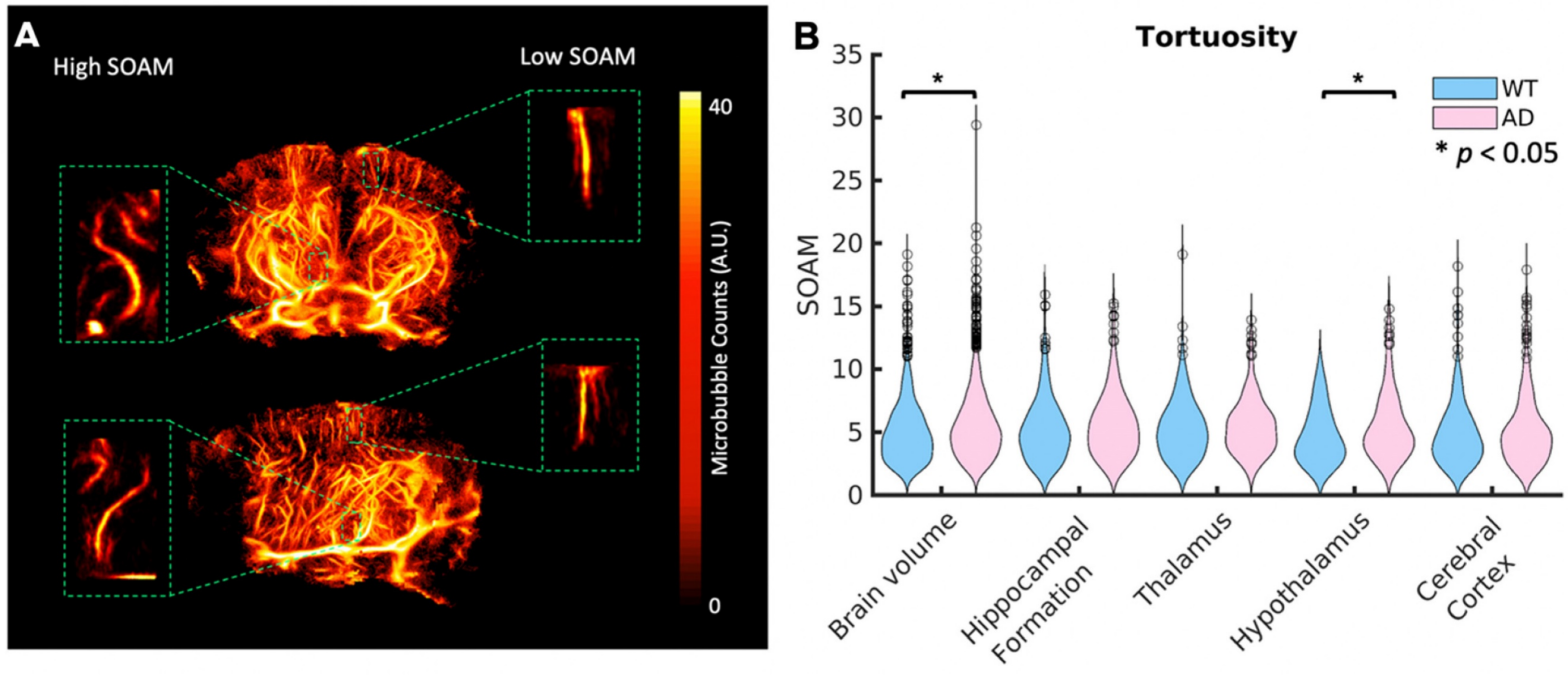
(A) A diagram showing volumetric views in the coronal and sagittal dimensions for the second WT mouse in Fig. 1. A zoomed in volume of a vessel with high tortuosity as calculated by the SOAM (left) and one with low tortuosity (right) are shown in the green boxes. (B) Violin plots showing the tortuosity per vessel across the whole brain volume and each of the four selected regions for WT and AD mice. Statistical significance (*α* = 0.05) was determined using Kruskal-Wallis one-way ANOVA tests, represented by a *. The AD mice had significantly higher SOAM metric values than WT mice across the brain volume and in the hypothalamus.

A*β* plaques are known to increase tortuosity in the microvasculature. To compare tortuosity between groups, MIPs showing the SOAM metric projected onto the skeletonization of the vasculature are shown in the last row of Fig. 3 for the whole brain volume and Fig. 4 for the four selected regions of interest. Unlike velocity and blood flow, there do not appear to be large differences in values between regions. In Fig. 7 A, a coronal and sagittal view of a 3D rendering of a ULM image is shown. A green box shows the zoomed in rendering of a with a high SOAM on the left and one with a low SOAM on the right. To compare the tortuosity between groups, violin plots are shown in Fig. 7 B for the brain volume and selected regions. When comparing the SOAM in WT and AD mice, there is a significant difference in total tortuosity between the two groups (p = 0.002, WT = 5.0 *±* 2.5, AD = 5.6 *±* 2.7), with a significant difference in the hypothalamus (p = 0.01, WT = 4.7 *±* 2.2, AD = 5.6 *±* 2.7), one of the areas affected in early stages of AD, where the AD group has higher tortuosity, as measured by the SOAM, than the WT group. The effect size across the brain volume is 0.2 and in the hypothalamus is 0.3. This implies that while there is a relationship, it is weak. There are no significant differences in the other three regions.

## Discussion

Here, non-invasive volumetric ULM imaging has been performed on mice with and without AD. APP^NL-G-F^ Knock-in mice have been reported to experience a reduction in CBF, as measured by fMRI, due to AD. However, the underlying mechanisms behind this reduction in CBF are unknown. To demonstrate the feasibility of using volumetric ULM to non-invasively investigate these mechanisms, metrics to investigate the differences between AD and WT mice, including diameter, velocity, blood flow, and tortuosity, were compared, with significant differences in tortuosity and blood flow vs vessel diameter between groups.

To determine if this technique is sensitive to changes in tortuosity between groups, a documented vascular change due to AD, the SOAM was used. The SOAM tortuosity metric was used as previous studies have shown that the SOAM is a more accurate tortuosity metric, especially in 3D, than the distance metric for example, due to its ability to distinguish tightly coiled vessels 39.

There was a significant difference between groups in tortuosity (p = 0.002), as calculated by the SOAM, across the whole brain volume. There was also a significant difference in the hypothalamus (p = 0.01), a region containing the mammillary bodies, which is one of the earliest regions affected by AD. These relationships have low p-values, but they also have low effect sizes. Future studies with larger sample sizes could provide more insight into the strength of this trend. Previous studies have shown higher tortuosities in the hippocampus 18, however, here there was not a significant difference between groups (p = 0.06). A different mouse model was used in the previous study, but a larger sample size would be needed to determine if this mouse model experiences differences in tortuosity in this region.

Previous results have shown a significant difference in velocity between AD and WT mice 18. In our comparison, there were no significant difference between groups in diameter (p = 0.29). There was however a significant increase in velocity in mice with AD (p = 0.002). A decrease in velocity is a known symptom of AD and has been shown in studies using different mice models 18. However, this relationship has a very low effect size (d = 0.1). Several factors could be contributing to this difference in velocity. First, previous studies have been performed post craniotomy, which could affect the cerebral hemodynamics. This study is completely non-invasive, removing any effects caused by animal response to surgery. Second, we were imaging transcranially on mice up to 11 months old. Previous non-invasive volumetric ULM has only been performed on rodents up to 10 weeks of age 21, 40 when the skull is much softer. Therefore, we have more shadowing, reflections, and attenuation than is present in younger mice or in previous studies with a craniotomy 18. This shadowing is primarily present in the cortex, where smaller vessels typically exist. Therefore, if the differences in velocity are primarily seen in smaller cortical vessels, we would not be as sensitive to these differences. Third, we used the APP^NL-G-F^ Knock-in AD mouse model, which forms A*β* plaques, but other vascular changes, including changes in velocity, have not yet been documented as the disease progresses.

Decreases in cerebral blood flow of 21% in 8-month-old mice, when measured by fMRI, have been reported 41, 42, but the mechanisms behind these changes, including investigation into vessel diameter and velocity, have not been reported. fMRI values are also based on BOLD signal, which is an indirect measure of blood flow. Future studies could use a different, more widely studied, vascular model of AD to provide more insight about the vascular changes seen during AD progression. Fourth, these calculations were based on the mean, and do not take into account differences in certain vessel sizes. The relationship between velocity and diameter is complex and depends on the type of vessel 43. There is, however, a better-defined relationship between diameter and blood flow. There was an increase across the brain volume in blood flow (p = 0.04); however, again, there was a very small effect size (d = 0.09). When comparing across all vessels, there was a significant difference in the diameter vs blood flow power log relationship.

The slope of the linear relationship between diameter and blood flow is equivalent to the exponent of the exponential curve showing the relationship between these two variables. Therefore, the higher this linear log slope, the faster the exponential curve grows between these two variables. This implies that for vessels of the same size, a larger slope would mean that there would be a higher blood flow. There was a significant difference in this slope between WT and AD mice across the whole brain volume (p = 0.02) and the hippocampal formation (p = 0.05). Both of these have very large effect sizes, with a Cohen's d over -2. The slope for WT mice was similar to the ideal and existing literature for mice 20, 38; however, in AD mice, it was lower than typically expected. This could be due to multiple mechanisms including increases in blood viscosity and vessel stiffness caused by AD or differences in the development of the arteriolar tree due to A*β* plaques.

In the ULM images, some cortical areas do not have as much vessel filling as other, deeper brain regions, leading to lower than expected vessel densities. Previous studies show a higher vessel density, calculated as the total length of vessels per volume in the cortex 44, 45. However, since these vessels are smaller in diameter, this could be why our percent coverage metric is smaller in this region. A smaller vessel density in the cortical region could be due to shadowing caused by the skull. Previous studies show that the mean capillary diameter is around 11 *µ*m while venule diameter is around 9 *µ*m in mice using 2-photon fluorescence microscopy 46. These are both lower than our minimum diameter. Therefore, information about the smallest vessels is missing in our comparison. However, our values align with previous transcranial ULM studies 20. There was a significant difference in the vessel density between groups across the whole brain volume (p = 0.02), where AD mice had higher vessel filling than WT mice. The AD mice weighed less than the WT (p = 0.001), while the MB concentration was scaled by weight, the infusion rate was not, so there may have been more MBs present in the blood stream. However, no significant differences were detected between groups in the four selected brain regions. The cerebral cortex had lower vessel filling percentages than other regions in both groups, likely due to shadowing from the skull. To better analyze those cortical regions, methods to improve vessel filling, such as longer acquisition times, and methods to improve imaging contrast, such as higher transmit MIs (through focusing or higher driving voltages), more angular compounding, or better transcranial imaging windows (through angled transducer orientations, for example). The skull introduces more challenges to ULM imaging, but removing the invasive nature of previous studies creates the potential for serial imaging without requiring surgery, increasing the capabilities of ULM for investigating AD pathology. The volumetric component of this imaging allows for a much larger field of view containing whole brain regions, which is not possible with just 2D slices. This provides a more precise study of the brain and should improve velocity and tortuosity calculations due to the continuous presence of all of the vessel in 3D. Previous ULM studies have seen much lower average velocities and SOAM values in both WT and AD mice than our values 18. However, this study was performed in 2D and with a craniotomy. In 2D, vessels can move in and out of the frame based on curvature, which bias these quantification metrics. Our ULM images also had a lower resolution, as calculated using FSC, than our smallest calculated vessel diameter. The FSC resolution measurement is a resolution estimate based on the whole image, but it is limited in regions of low signal and in undersampled images. However, it is a commonly used metric to calculate resolution in ULM images.

All of our comparisons rely on the skeletonization of the ULM images. This relies on the extraction of centerlines from the binarized image. Since the image is binarized, some vasculature may inherently be removed, particularly smaller vessel sizes. Additionally, centerline extraction can be difficult in ULM images due to the nature of the technique, where microbubbles tracks are used 47. The skeletonization could be introducing some noise in areas where it either detects vessels that are not there or misses vessels. However, for all of our metrics, we used both the skeletonization and the tracked ULM results, reducing the likelihood of including vessels that do not exist. Due to the presence of the skull, larger, deeper vessels are favored in our images, so there could be some information in smaller cortical vessels missing. In some of the projections on to the skeleton, the velocity and flow are sparser than the diameter. This is due to velocity calculations that were zero. This could be due to a lack of moving MBs in that region or an artifact of taking the mean velocity over time in sparsely travelled vessels. The skeletonization for one AD mouse was also missing vessels in the parietal cortex, only detecting two vessels. This could be due to increased skull shadowing in this mouse, since the parietal cortex is closer to the skull. The mice may have had slightly different orientation or position, contributing to greater shadowing in this region.

In this study, we compared five WT and five AD mice using non-invasive, volumetric ULM, a comparison that has only previously been performed in 2D, after an invasive removal of the skull via craniotomy. In the future, this study will be repeated with increased sample size to establish statistical significance of the trends suggested by this data. Additionally, future studies including multiple models of Alzheimer's Disease that include different components of this disease, like the development of neuron tangles, would provide more insight into this disease and how its different components affect the microvasculature. Without histology, the exact regions of degeneration could not be confirmed; however, our results are consistent with expectations from the existing literature and the specific mouse model and illustrate the power of non-invasive ultrasound imaging for assessing vascular changes in neurodegenerative disease models. In future studies, histology could be performed in conjunction with ULM to validate and augment the ULM results.

## Conclusions

This research demonstrates that volumetric super-resolution ultrasound imaging can non-invasively detect microvascular differences in the brain caused by A*β* plaques between AD and WT mice. Differences in tortuosity were detected across the brain volume and in the hypothalamus, a region that form A*β* plaques in this mouse model. Differences in the linear log slope between the diameter and blood flow were also detected, with AD mice having a smaller slope than WT mice across the brain volume and in the hippocampus. The non-invasive nature of this technique provides the potential to perform longitudinal vascular comparisons as AD progresses. This allows for better study into vascular changes during different stages of the disease, providing more insight into the nature, effects, and causes of this microvascular degeneration.

## Supplementary Material

Supplementary figure and table.

## Abbreviations

AD: Alzhiemer's disease
A*β*: amyloid beta
APP: amyloid beta precursor protein
FSC: fourier shell correlation
MIP: maximum intensity projection
SVD: singular value decomposition
SOAM: sum of angles metric
ULM: ultrasound localization microscopy
WT: wildtype
