# Supplementary material for: Non-invasive volumetric ultrasound localization microscopy detects vascular changes in mice with Alzheimer's disease
Source: Theranostics. 2025 Jan 1;15(3):1110–21. doi: 10.7150/thno.99097 (PMC11700853; doi:10.7150/thno.99097)
Supplement: Supplementary file 1 — Supplementary figure and table. [file thnov15p1110s1.pdf]

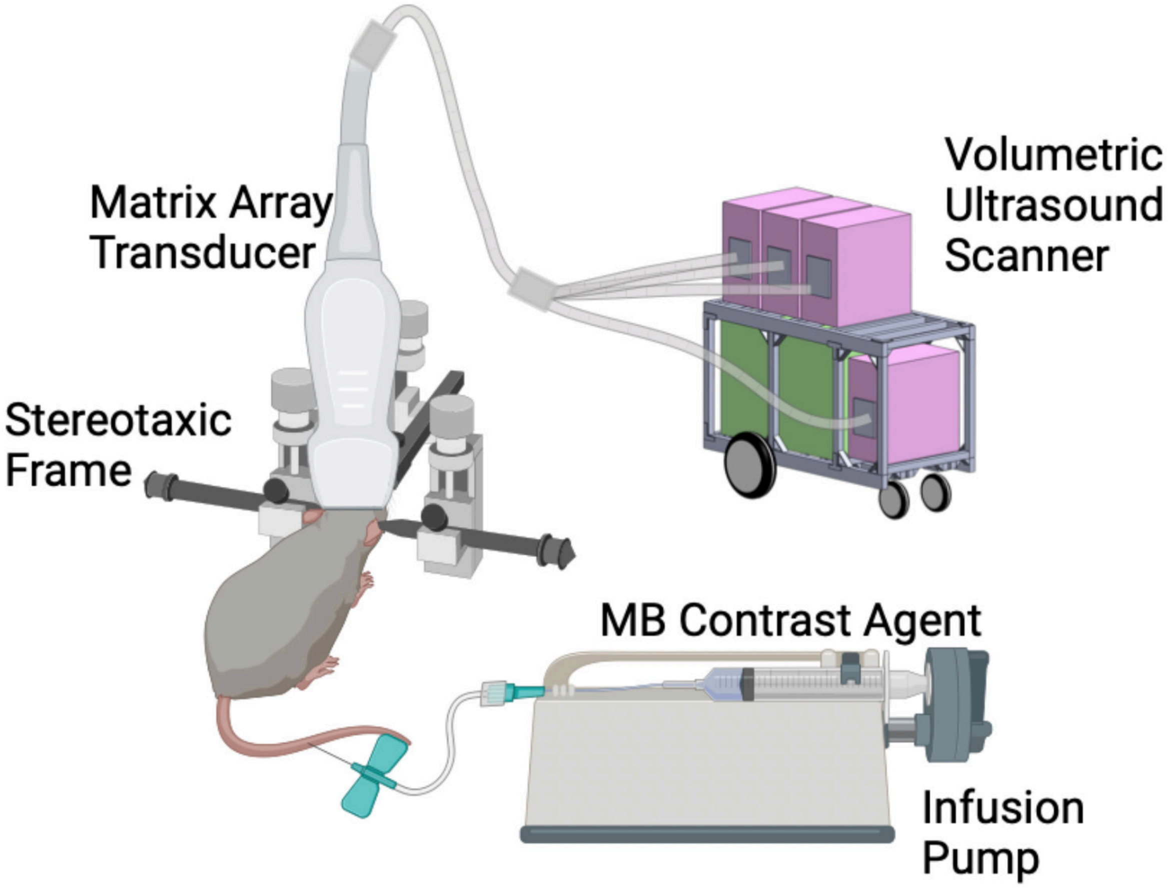

Figure S1: Diagram of the experimental setup showing the mouse placed in a stereotaxic frame. An ultrasound matrix array transducer is placed on the depilated head over a layer of ultrasound imaging gel, transmitting at 7.81 MHz controlled by a 1024-channel volumetric system. Microbubbles are continuously infused through the tail vein. Made with BioRender.

Table S1: Statistical *p*-values resulting from Kruskal-Wallis one-way ANOVAs performed between wildtype mice and mice with Alzheimer's Disease for all compared parameters in all compared regions, with effect sizes calculated using Cohen's d. Significant values are shown in bold.

	**Brain Volume**	**Hippocampal Formation**	**Thalamus**	**Hypothalamus**	**Cerebral Cortex**
Vessel Density	***p*=0.02**<br>**d=2.53**	*p*=0.12<br>d=1.17	*p*=0.92<br>d=0.03	*p=0.17*<br>*d=0.86*	*p*=0.75<br>d=0.33
Diameter	*p*=0.29<br>d=0.03	*p*=0.86<br>d=-0.01	*p*=0.83<br>d=-0.01	*p*=0.74<br>d=-0.06	*p*=0.39<br>d=-0.04
Velocity	***p*=0.002**<br>**d=0.10**	*p*=0.29<br>d=0.11	*p*=0.78<br>d=-0.06	*p*=0.46<br>d=0.10	*p*=0.10<br>d=0.12
Blood Flow	***p*=0.04**<br>**d=0.09**	*p*=0.38<br>d=0.12	*p*=0.48<br>d=0.08	*p*=0.56<br>d=-0.08	*p*=0.26<br>d=-0.14
Diameter vs Blood Flow	***p*=0.02**<br>**d=-2.04**	***p*=0.05**<br>**d=-1.72**	*p*=0.08<br>d=1.32	*p*=0.17<br>d=-0.84	*p*=0.12<br>d=-0.94
Tortuosity	***p*=0.0002**<br>**d=0.21**	*p*=0.06<br>d=0.14	*p*=0.27<br>d=0.07	***p*=0.01**<br>**d=0.34**	*p*=0.21<br>0.11
